# Admission C – reactive protein after acute ischemic stroke is associated with stroke severity and mortality: The 'Bergen stroke study'

**DOI:** 10.1186/1471-2377-9-18

**Published:** 2009-04-28

**Authors:** Titto T Idicula, Jan Brogger, Halvor Naess, Ulrike Waje-Andreassen, Lars Thomassen

**Affiliations:** 1 Department of Neurology, Haukeland Hospital, Bergen, Norway; 2 Institute of Clinical Medicine, University of Bergen, Bergen, Norway

## Abstract

**Background:**

There is growing evidence that inflammation plays an important role in atherogenesis. Previous studies show that C-reactive protein (CRP), an inflammatory marker, is associated with stroke outcomes and future vascular events. It is not clear whether this is due a direct dose-response effect or rather an epiphenomenon. We studied the effect of CRP measured within 24 hours after stroke onset on functional outcome, mortality and future vascular events.

**Methods:**

We prospectively studied 498 patients with ischemic stroke who were admitted within 24 hours after the onset of symptoms. CRP and NIH stroke scale (NIHSS) were measured at the time of admission. Short-term functional outcome was measured by modified Rankin scale (mRS) and Barthel ADL index (BI) 7 days after admission. Patients were followed for up to 2.5 years for long-term mortality and future vascular events data.

**Results:**

The median CRP at admission was 3 mg/L. High CRP was associated with high NIHSS (p = 0.01) and high long-term mortality (p < 0.0001). After adjusting for confounding variables, high CRP remained to be associated with high NIHSS (p = 0.02) and high long-term mortality (p = 0.002). High CRP was associated with poor short-term functional outcomes (mRS > 3; BI < 95) (p = 0.01; p = 0.03). However, the association was not significant after adjusting for confounding variables including stroke severity (p = 0.98; p = 0.88). High CRP was not associated with future vascular events (p = 0.98).

**Conclusion:**

Admission CRP is associated with stroke severity and long-term mortality when measured at least 24 hours after onset. There is a crude association between high CRP and short-term functional outcome which is likely secondary to stroke severity. CRP is an independent predictor of long-term mortality after ischemic stroke.

## Background

There is growing evidence that C-reactive protein (CRP), a peripheral marker of inflammation, is also a marker of generalized atherosclerosis [1]. This relationship between inflammation and atherosclerosis make CRP a potential marker for prognosis after vascular events and a potential predictor of future vascular events.

Large population-based studies show that high CRP is a risk factor for future cardiovascular events [2-4]. The recent JUPITER trial shows that the use of rosuvastatin in patients with high CRP has a significant impact both in reducing the CRP level and in lowering future vascular events [5]. This indicates the role of inflammation in atherogenesis and suggests that CRP can be used as marker of future events.

The role of CRP as a marker during and after ischemic stroke is less extensively studied in comparison to coronary artery disease. The Rotterdam study shows that although high CRP is associated with the risk for future stroke, it is not useful for individual stroke prediction [6]. On the other hand the Framingham study shows that high CRP is associated with a greater risk for ischemic stroke or TIA [7]. Studies in patients who already had a stroke shows an association between high CRP and stroke presentation, outcomes and future vascular events [8-14]. The sample sizes in these studies, however, have been relatively modest, and follow-up usually short (3–12 months). We conducted a collaborative review of 11 studies published between 1999 and 2004. There was substantial variation in sample sizes (range 37–716), study design (prospective, retrospective, controls included), subtype of stroke included and main outcome measures [8-12,14-19]. Moreover, the results from some studies were negative. In their pivotal review, Di Napoli et al concluded that there is insufficient evidence to justify the routine use of CRP for either primary or secondary risk stratification for cerebrovascular disease alone [20]. In addition, only few studies have analyzed the relationship between elevated admission CRP levels and stroke severity or stroke etiology. In short, there is need for more studies to clarify the exact role of CRP in cerebrovascular disease.

The aim of this study was to help clarify the role of early CRP in ischemic stroke by determining its association with stroke severity, stroke etiology, functional outcome, mortality and future vascular events, in a larger study than most of the previous studies.

## Methods

Data for this prospective study was obtained at Haukeland University Hospital, Bergen, Norway, which serves a well-defined population of 235,000. The study was done as a part of a cohort study (The Bergen stroke study) in which data was collected from all ischemic stroke patients admitted to the department of neurology from February 2006 to September 2008. Patients who were admitted more than 24 hours after the onset of symptoms were excluded. Patients who were found with stroke or awoke with stroke were included if it was known that the patient had been normal <24 hours before blood sampling. Patients whose clinical and radiological findings were not consistent with stroke were excluded from the study.

At admission, plain CT scan of the head was done to rule out haemorrhage. Blood samples for CRP were taken at the time of admission, and analyzed by Tina-quant latex method using Modular P (Roche Diagnostics). The NIH stroke score (NIHSS) was assessed by a neurologist at the time of admission. The NIHSS was categorized as 0–7, 8–14 and >14. Eligible candidates were given intravenous thrombolysis according to SITS-MOST protocol [21].

Risk factors were defined according to protocol. Hypertension (HTN) was defined as treatment with antihypertensive drugs before stroke onset or introduction of antihypertensive drugs before discharge. Diabetes mellitus (DM) was defined as treatment with glucose lowering medications or diet prior to stroke onset, or a fasting serum glucose >7.7 mmol/L during hospital stay. Smoking was defined as the use of at least one cigarette per day prior to stroke onset.

Based on the clinical presentation of stroke, patients were classified according to the Oxford Community Stroke Project (OCSP) classification [22]. The etiology of stroke was classified according to TOAST criteria [23]. Clinical details such as physiologic parameters, type of treatments and radiographic findings were registered. Age was categorized as <50, 50–59, 60–69 and ≥70 years. Outcome was measured by modified Rankin Scale (mRS) and Barthel ADL index (BI) 7 days after stroke onset. Poor outcome was defined as mRS >2 or BI <95.

Survival data was obtained from the National Population Registry of Norway, where all the permanent residents are registered. Admissions for acute vascular events after the index stroke admission were obtained from searches of the electronic discharge diagnosis registry at the hospital using ICD10 codes I20, I21, I22, I63, G45 (excluding G454), I61, I74. These electronic events were then manually validated by two of the authors (JB, TI) and the time to first future event of each type ascertained. Approximately half of the electronic discharge registry events were false positives because of the re-use of the acute vascular event diagnosis when the "sequelae of" should have been used.

Informed consent was obtained from all patients. The study was approved by the local ethical committee.

### Analysis

Due to the skewed distribution of CRP, we chose somewhat arbitrary cut points for CRP of 3 and 10 mg/dl, corresponding approximately to the median (50%) and upper 20% distribution. The 3 mg/dl cut point corresponds to the "high" CRP level in previous primary prevention studies [24].

First, the prevalence of an elevated CRP was studied in various groups such as pre-stroke demographic factors, pre-existing illness, risk factors for stroke, stroke severity and time to hospital. A multivariate analysis was then performed to identify independent predictors of an elevated CRP.

To study the association of CRP with stroke severity, etiology and outcomes we performed the following analysis. The crude association of categories of CRP with stroke severity (NIHSS and OCSP), etiology (TOAST), functional outcome (mRS and BI), mortality and future vascular events was computed. The Pearson χ^2 ^test was used for count table data, and the log rank test used for survival-type tables. For the adjusted analysis, logistic regression was used for most analyses. For dependent variables with two outcome levels, ordinary logistic regression was. For dependent variables with more than two outcome levels, multivariate logistic regression was performed. For survival-type outcomes, Cox regression was used. For dependent variables except stroke severity, we adjusted for age, sex, diabetes, thrombolysis and stroke severity (NIHSS). When stroke severity was the dependent variable, adjustment was done for age, sex, thrombolysis and the presence of DM.

## Results

During the inclusion period, 743 patients with ischemic stroke were registered. Of these, 238 were excluded because the interval between stroke onset and blood sampling was >24 hours. Of the remaining, seven did not have an admission CRP, leaving 498 patients for the present analysis. The population characteristics with regards to important pre-stroke risk factors, stroke presentation and outcomes are shown in Table 1. The mean age (SD) of the study population was 69.3 (14) years. Men, on average, were 7.5 years younger than women. The distribution of CRP (mg/L) values was highly skewed (median 3, 90% percentile 18), with a somewhat higher geometric mean value in women (3.7) than in men (3.1) (p = 0.08).

**Table 1:** Sample size, distribution of risk factors, stroke presentation and outcomes in the 'Bergen stroke study' (n = 498) by sex

		**Men**	**Women**	**Total**
**Sample size**		302	196	498
***Risk factors***				

Age – mean (SD)		66.4	73.9	69.3
Total cholesterol – mean		5.04	5.91	5.39
Smoking – current smokers (%)		28	19	24
Hypertension (%)		46	57	51
Pre-existing diabetes mellitus (%)		13	13	13
Pre-existing cerebrovascular disease (%)		30	26	29
Pre-existing coronary artery disease (%)		25	16	21
Pre-existing cerebrovascular or coronary artery disease (%)		44	34	40
**Stroke presentation**				

Time to blood test (%)	<3 hours	38	35	37
	3–6 hours	15	19	17
	6–12 hours	12	11	12
	12–24 hours	10	7	9
	Awoke with Stroke (<24 hours)	16	14	15
	Other (<24 hours)	7	11	8
OCSP classification (%)	LACI	21	31	25
	TACI	15	21	17
	PACI	45	35	41
	POCI	19	13	17
NIHSS (%)	<7	74	68	72
	7–13	13	16	14
	14+	11	12	11
Treated with i.v. thrombolysis (%)		20	17	19

NIHSS = National Institute of Health Stroke Score

OCSP = Oxford Community Stroke Project Classification

LACI = Lacunar infarct

TACI = Total Anterior Circulation Infarct

PACI = Partial Anterior Circulation Infarct

POCI = Posterior Circulation Infarct

In table 2, we show the prevalence of an elevated CRP according to various potential predictors, such as age and sex, pre-existing illnesses, such as DM and coronary artery disease, stroke severity and time to hospital. In univariate analysis, only stroke severity (NIHSS, OCSP) and pre-existing diabetes were significant. In a multivariate model including one measure of stroke severity (NIHSS) and pre-existing diabetes, an elevated CRP was independently predicted both by stroke severity and pre-existing diabetes (p = 0.03; p = 0.04).

**Table 2:** Prevalence of an elevated CRP (>10 mg/L) in various patient subgroups in the 'Bergen stroke study' (n = 498)

**Prevalence of elevated CRP (>10 mg/L)**					
**Group**		**%**	**Univariate p-value**	Multivariate OR *	Multivariate p-value**

Age	<50	14	0.49		
	50–59	13			
	60–69	13			
	70+	18			

Sex	Male	14	0.28		
	Female	18			

NIHSS	<7	13	0.015	(ref)	0.03
	7–13	25		2.15	
	14+	21		1.89	

OCSP classification	LACI	15	0.005		
	TACI	26			
	PACI	10			
	POCI	19			

Prior Cerebrovascular disease	No	16	0.97		
	Yes	16			

Pre-existing diabetes mellitus	No	14	0.01	(ref)	0.04
	Yes	27		1.96	

Smoking habits	Never smoker	19	0.29		
	Former smoker	14			
	Current smoker	12			

Time to hospital	<3 hours	13	0.20		
	3–5.9 hours	20			
	6–11.9 hours	10			
	12–23.9 hours	18			
	Woke up with Stroke (<24 hours)	17			
	Other (<24 hours)	26			

NIHSS = National Institute of health Stroke Score

OCSP = Oxford Community Stroke Project Classification

LACI = Lacunar infarct

TACI = Total Anterior Circulation Infarct

PACI = Partial Anterior Circulation Infarct

POCI = Posterior Circulation Infarct

* Odds ratio for the variables that were significant on multivariate stepwise logistic regression analysis.

** p-value for the variables that were significant on multivariate stepwise logistic regression analysis.

In Table 3 we show the association of CRP with stroke severity, etiology, short-term outcome, mortality and future vascular events. An elevated CRP was associated with increased stroke severity (NIHSS, OCSP) (p = 0.01, p = 0.006). Seventy nine percent of the low-CRP group had a mild stroke (NIHSS<7), whereas only 60% of the high-CRP group had a mild stroke. The most common stroke subtype in the high CRP group was TACI (29%), whereas in the low and medium CRP groups the most common presentation was PACI (41% and 49%). The likely etiology of stroke on the TOAST classification was statistically significantly associated with CRP (p = 0.04). The high CRP group had a higher frequency of stroke of cardioembolic origin (38% in the high CRP group vs. 21% in the low CRP group). CRP was associated with short-term functional outcome on mRS (p = 0.04), with the high CRP group having a 44% risk for a poor outcome vs. 26% for the low CRP group. CRP was associated with short-term functional outcome on BI (p = 0.03), with the high CRP group having a 40% risk for poor outcome vs. 24% for the low CRP group. CRP was not associated with future hospital admissions for vascular events (p = 0.98).

**Table 3:** Association of categories of admission CRP with two different measures of stroke severity (NIHSS, OCSP), stroke etiology (TOAST), stroke outcomes (mRS, BI, mortality) and future vascular events in the 'Bergen stroke study' (n = 498)

		CRP in Categories*			
		**<3**	**3–9.9**	**> = 10**	**p-value**
NIHSS	<7	79	73	60	0.01
	7–13	10	16	24	
	14+	11	11	16	
	*Total*	100	100	100	

OCSP	LACI	25	25	25	0.006
	TACI	16	14	29	
	PACI	41	49	26	
	POCI	19	12	20	
	*Total*	100	100	100	

TOAST	Atherosclerosis	11	16	11	0.04
	Cardioembolic	21	28	38	
	Small Vessel Disease	17	16	13	
	Other	3	2	5	
	Unknown	49	38	34	
	*Total*	100	100	100	

BI	*>95*	76	71	60	0.03
	*<95*	24	29	40	
	*Total*	100	100	100	

mRS	0–1	49	44	33	0.04
	2	25	25	24	
	3–6	26	31	44	
	*Total*	100	100	100	

Mortality	30-day	4	5	9	9.5E-08
	6 months	4	11	20	
	12 months	5	13	31	
	2 years	8	15	44	

Future Vascular Events	30-day	3	3	2	0.98
	6 months	8	6	5	
	12 months	11	9	11	
	2 years	12	12	17	

NIHSS = National Institute of Health Stroke Score

OCSP = Oxford Community Stroke Project Classification

TOAST = Trial of Org 10172 in Acute Stroke Treatment

mRS = Modified Rankin scale

BI = Barthel index

* Percentages within each CRP categories are shown columns

**Figure 1:**
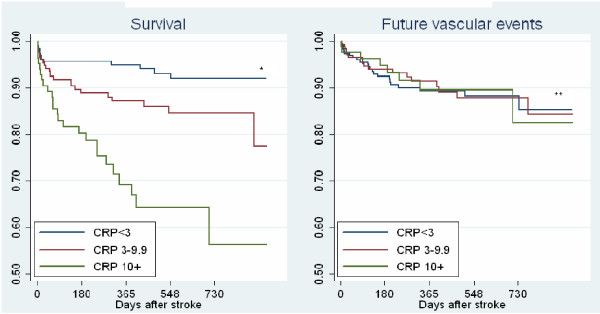
**Kaplan-Meier curve for survival and future vascular events after ischemic stroke based on admission CRP in categories(n = 498)**. *p-value = < 0.001. **p-value = 0.98.

Since CRP was associated with stroke severity and the pre-existing DM, and the patient sample spanned a wide age range, we wanted to see if the effect of CRP on outcomes was independent of stroke severity, age, gender, effect of intravenous thrombolysis and presence of DM. Both the unadjusted (equivalent to table 3) and the adjusted multinomial analysis are shown in Table 4. The unadjusted analysis showed a significant association with stroke severity, functional outcomes, mortality and etiology. Stroke severity was associated with admission CRP even after adjusting for age, sex, intravenous thrombolysis and the presence of DM. After adjustment, CRP was predictive of mortality but not for functional outcomes. Figure 1, shows the event curves for survival and future vascular events. The survival was poorer in the high CRP group not only immediately after stroke, but also for our observation period of 2.5 years.

**Table 4:** Unadjusted and adjusted association between CRP and stroke severity (NIHSS, OCSP), stroke etiology (TOAST), outcomes (mRS, BI, mortality) and future vascular events in the 'Bergen stroke study' (n = 498)

**Subgroup**			**Unadjusted**			Adjusted*		
**CRP categories:**			**3–9.9**	**10+**	**p**	**3–9.9**	**10+**	**p**
NIHSS**	7–13	OR	1.77	3.17	0.01	1.85	3.28	0.02
	14+	OR	1.11	1.89		1.03	1.85	

OCSP (reference: LACI)	TACI	OR	0.84	1.79	0.008	0.87	1.61	0.03
	PACI	OR	1.15	0.63		1.23	0.63	
	POCI	OR	0.64	1.07		0.74	1.21	

TOAST (reference: Athero-thrombotic)	Cardioembolic	OR	0.90	1.89	0.04	0.93	2.03	0.09
	Small vessel	OR	0.67	0.81		0.67	1.06	
	Other	OR	0.38	1.65		0.49	3.86	
	Unknown	OR	0.53	0.73		0.53	0.90	

Poor outcome (mRS >2)		OR	1.2	2.2	0.01	1.04	1.07	0.98
Poor outcome (BI <95)		OR	1.26	2.08	0.03	1.02	1.18	0.88
Mortality		HR	2.20	5.49	1.20E-06	2.31	3.47	0.002
Future Vascular Events		HR	0.95	0.95	0.98	0.86	0.84	0.87

NIHSS = National Institute of health Stroke Score

OCSP = Oxford Community Stroke Project Classification

TOAST = Trial of Org 10172 in Acute Stroke Treatment

mRS = Modified Rankin scale

BI = Barthel index

HR = Hazards ratio from Cox regression

OR = Hazards ratio from logistic regression

*Adjusted for age, sex, NIHSS, pre-existing diabetes and intravenous thrombolysis in categories

**Adjusted only for age and sex in categories

## Discussion

Our study in ischemic stroke patients shows that a high CRP at admission is associated with more severe stroke, cardioembolic etiology, poor functional outcome and high mortality. After adjusting for the effect of confounding factors, high CRP remained to be associated with more severe stroke and high mortality, but not with functional outcome or etiology. CRP was not associated with future vascular events.

The association between high CRP and a high stroke severity remains unexplained. There is a distinct possibility that elevated CRP is a direct response to the extent of cerebral tissue injury [25]. But as an inflammatory marker, it is also possible that high CRP is associated with underlying processes that cause a more severe stroke. A small number of papers with a relatively small number of patients have studied the time course of CRP after stroke onset [19,26-28]. Some of them have found an increase in CRP after stroke. The level of increment had been either very small – about one unit increase [19,27,28], or a very large increase (10 units increase in 24 hours) [26]. These discrepancies, too, remain unexplained. Whether high CRP causes more severe stroke or vice versa needs to be further studied in population-based larger cohort studies, or in clinical studies with very early and closely spaced repeated measurements.

The prognostic significance of early CRP after stroke has significant clinical implications. Previous stroke studies have shown an association between high CRP and poor outcome [8,10,11,13,14,29]. Our study showed that the long-term mortality was significantly associated with CRP even after adjusting for age, sex and stroke severity. We measured long-term mortality over a period of 2.5 years. Since we did not have data on the causes of death, we cannot be certain that the high mortality in patients with elevated CRP is due to vascular events. If most of the death were secondary to a vascular event, we would anticipate a similar association between CRP and future vascular events. However, this was not found in our study. The results from a previous study also shows that CRP is associated with the risk of death but not with vascular death or future vascular events [12]. In clinical practice we may consider high CRP as a "red flag" marker of high mortality, but the therapeutic implications of this finding remain uncertain.

The positive association between high CRP and cardioembolic etiology is rather intricately related to stroke severity. Stroke secondary to cardioembolism tends to have a higher stroke severity. In our study the mean NIHSS of stroke secondary to cardioembolism (7.7) was higher than large-artery (4.2), small-vessel (3.1), and unknown (5.1). This might explain why the association between high CRP and cardioembolism was no more significant after adjusting for confounding factors including stroke severity. Apart from that, a large proportion of patients in our study (42%) had unknown etiology which may add further uncertainty.

Our study, in which CRP was measured within 24 hours after stroke onset, showed a crude association between high CRP and poor short-term functional outcome. However, when adjustment was done for confounding factors including stroke severity, the association was no more significant. We speculate that high CRP by itself is not damaging per se. It is probably an epiphenomenon for poor outcome. A recent large population genetics study suggests that CRP is partly genetically determined, and that a high CRP level is not damaging per se [30].

Studies in patients free of vascular disease at baseline show a positive association between high CRP and future vascular events [31-33], We failed to show such an association in stroke patients when CRP was measured within 24 hours after stroke onset. A few previous studies were done in which CRP was measured early after stroke onset (within 12 hours) [15,19,34]. Two of those studies failed to show any association between CRP and future vascular events [15,19], but one of them showed an association between high CRP and future vascular events [34]. Chronic inflammation is considered to be a risk factor for future vascular events [35]. Baseline CRP in the absence of obvious causes like infection or inflammation may represent the chronic inflammatory status of a patient. CRP level after the acute phase of stroke, however, is confounded by many factors including index stroke severity, co-existing infections, stress and complications of stroke such as deep vein thrombosis [14].

Some of limitations of our study must be acknowledged. CRP was measured only at one time point (at admission). Single CRP measurement can be influenced by many factors such as infections, stress, timing of measurement and lab error. Serial measurements of CRP were not available because this was not considered during the planning of the epidemiological cohort that this study arises from. Another limitation of the study was the un-availability of a cause-specific survival data. Data on mortality from vascular events only, would have been more ideal as this would directly link the role of CRP in stroke outcome. Another limitation of this study was the substantial heterogeneity in stroke, and the subsequent necessity of a relatively large sample size to detect effects. However, compared to the previous studies, this is a relatively large study. Given that the etiology of stroke is complex, the sample size problem is expected.

## Conclusion

A high admission CRP in ischemic stroke patients is clearly associated with more severe stroke and high long-term mortality. The clinical implications of these findings are unclear at present. It remains to be seen whether CRP is a marker of stroke severity, or is a response to stroke, or a mixture of both.

## Competing interests

The authors declare that they have no competing interests.

## Authors' contributions

TTI and JB participated in the design of the study, participated in analysis and interpretation of data, participated in manuscript drafting. UWA participated in data collection, participated in revising manuscript. HN participated in the design of the study, participated in data collection. LT participated in the design of the study, participated in data collection, participated in manuscript drafting.

## Authors' Information

TTI:

Qualification: M.D,

Position: Vascular Neurologist, Research Fellow, University of Bergen, Bergen, Norway

JB:

Qualification: M.D, PhD,

Position: Neurophysiology Fellow, Postdoctoral Fellow, University of Bergen, Bergen, Norway

UWA:

Qualification: M.D, PhD.

Position: Vascular Neurologist, Haukeland University Hospital, Bergen, Norway

HN:

Qualification: M.D, PhD

Position: Vascular Neurologist, Haukeland University Hospital, Bergen, Norway

LT:

Qualification: M.D, PhD

Position: Head of Vascular Neurology, Haukeland Univesrity Hospital, Bergen, Norway

## Pre-publication history

The pre-publication history for this paper can be accessed here:


